# Toll-Like Receptor 1 Locus Re-examined in a Genome-Wide Association Study Update on Anti–*Helicobacter pylori* IgG Titers

**DOI:** 10.1053/j.gastro.2022.01.011

**Published:** 2022-01-12

**Authors:** Suk Yee Lam, Michiel C. Mommersteeg, Bingting Yu, Linda Broer, Manon C. W. Spaander, Fabian Frost, Stefan Weiss, Henry Völzke, Markus M. Lerch, Ben Schöttker, Yan Zhang, Hannah Stocker, Hermann Brenner, Daniel Levy, Shih-Jen Hwang, Alexis C. Wood, Stephen S. Rich, Jerome I. Rotter, Kent D. Taylor, Russell P. Tracy, Edmond K. Kabagambe, Marcis Leja, Janis Klovins, Raitis Peculis, Dace Rudzite, Liene Nikitina-Zake, Girts Skenders, Vita Rovite, André Uitterlinden, Ernst J. Kuipers, Gwenny M. Fuhler, Georg Homuth, Maikel P. Peppelenbosch

**Affiliations:** 1 Department of Gastroenterology and Hepatology, Erasmus University Medical Center, Rotterdam, The Netherlands; 2 Department of Internal Medicine, Erasmus University Medical Center, Rotterdam, The Netherlands; 3 Department of Medicine A, University Medicine Greifswald, Greifswald, Germany; 4 Department of Functional Genomics, Interfaculty Institute for Genetics and Functional Genomics, University Medicine Greifswald, Greifswald, Germany; 5 Institute for Community Medicine, University Medicine Greifswald, Greifswald, Germany; 6 Division of Clinical Epidemiology and Aging Research, German Cancer Research Center, Heidelberg, Germany; 7 Network Aging Research, Heidelberg University, Heidelberg, Germany; 8 Framingham Heart Study, National Heart, Lung, and Blood Institute, Framingham, Massachusetts, USA; 9 Population Sciences Branch, Division of Intramural Research, National Heart, Lung, and Blood Institute, Bethesda, Maryland, USA; 10 USDA/ARS Children’s Nutrition Research Center, Baylor College of Medicine, Houston, Texas, USA; 11 Center for Public Health Genomics, University of Virginia, Charlottesville, Virginia, USA; 12 Institute for Translational Genomics and Population Sciences, Department of Pediatrics, Lundquist Institute for Biomedical Innovation at Harbor-UCLA Medical Center, Torrance, California, USA; 13 Laboratory for Clinical Biochemistry Research, University of Vermont College of Medicine, Colchester, Vermont, USA; 14 Division of Academics, Ochsner Health, New Orleans, Louisiana, USA; 15 Institute of Clinical and Preventive Medicine, Faculty of Medicine, University of Latvia, Riga, Latvia; 16 Latvian Biomedical Research and Study Center, Riga, Latvia; 17 Rīga Stradiņš University, Riga, Latvia

**Keywords:** Single-Nucleotide Polymorphism, Serology, Immunity, Bacteria

## Abstract

**BACKGROUND & AIMS:**

A genome-wide significant association between anti–*Helicobacter pylori (H pylori)* IgG titers and Toll-like receptor *(TLR1/6/10)* locus on *4p14* was demonstrated for individuals of European ancestry, but not uniformly replicated. We re-investigated this association in an updated genome-wide association study (GWAS) meta-analysis for populations with low gastric cancer incidence, address potential causes of cohort heterogeneity, and explore functional implications of genetic variation at the *TLR1/6/10* locus.

**METHODS:**

The dichotomous GWAS (25% individuals exhibiting highest anti–*H pylori* IgG titers vs remaining 75%) included discovery and replication sampls of, respectively, n = 15,685 and n = 9676, all of European ancestry. Longitudinal analysis of serologic data was performed on *H pylori*–eradicated subjects (n = 132) and patients under surveillance for premalignant gastric lesions (n = 107). TLR1/6/10 surface expression, *TLR1* mRNA, and cytokine levels were measured in leukocyte subsets of healthy subjects (n = 26) genotyped for *TLR1/6/10* variants.

**RESULTS:**

The association of the *TLR1/6/10* locus with anti–*H pylori* IgG titers (rs12233670; b = −0.267 ± SE 0.034; *P* = 4.42 × 10^−15^) presented with high heterogeneity and failed replication. Anti–*H pylori* IgG titers declined within 2–4 years after eradication treatment (*P* = 0.004), and decreased over time in patients with premalignant gastric lesions (*P* < 0.001). Variation at the *TLR1/6/10* locus affected TLR1-mediated cytokine production and TLR1 surface expression on monocytes (*P* = 0.016) and neutrophils (*P* = 0.030), but not mRNA levels.

**CONCLUSIONS:**

The association between anti–*H pylori* IgG titers and *TLR1/6/10* locus was not replicated across cohorts, possibly owing to dependency of anti–*H pylori* IgG titers on therapy, clearance, and antibody decay. *H pylori*–mediated immune cell activation is partly mediated via TLR1 signaling, which in turn is affected by genetic variation.

## Graphical Abstract

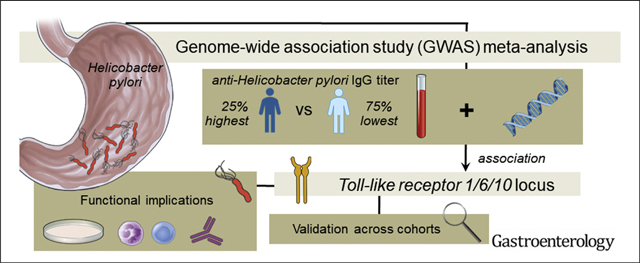

The discovery of *Helicobacter pylori* (*H pylori*) at the epithelial surface of the human stomach as late as 1983 represented a major breakthrough in gastric microbiology.^1^ This flagellated bacterium has since been implicated in the etiology of atrophic gastritis and gastroduodenal ulcerative disease,^2^ identified as a class 1 carcinogen for gastric cancer,^3–5^ and ranked as the most important contributor to infection-attributable cancers in 2018.^6^ With estimates indicating that more than half of the world’s population is colonized by *H pylori*, the size of this global health problem is further emphasized.^7^ Because *H pylori* gastric presence has been linked to early stages of gastric carcinogenesis according to the Correa model,^8^ eradication strategies have been implemented to prevent gastric cancer development.^9–12^ However, global resistance of *H pylori* to antibiotics is reaching alarming levels,^13^ which puts further pressure on the *H pylori*–related health burden and warrants new strategies to prevent colonization and infection-related consequences. It is generally accepted that *H pylori* infection is acquired during early childhood,^14–17^ but the overall rate of infection is reported to be much higher in developing countries.^18^ Although socioeconomic and environmental factors likely explain the wide variation in *H pylori* prevalence between regions and countries,^7^ genetic predisposition also needs to be considered. It has been shown that the same rearing environment contributes to a familial tendency to acquire *H pylori* infection, but higher similarity in monozygotic than dizygotic twin pairs indicates that genetic factors account for a large part of the variation.^19^ Some individuals are never infected by *H pylori*, and others are able to clear the infection spontaneously when colonized.^14,16^ Moreover, only a small proportion of the *H pylori*–colonized population develop gastric cancer,^20^ indicating that host-specific factors governing the host-pathogen interactions are involved in disease risk.^21^ Because the host genetic background is suggested to be involved in the clinical outcome of *H pylori* infection, ^22^ a better understanding of the genetic contributions to the interaction between host and *H pylori* may improve our insight into this complex relationship.

An increasing number of genome-wide association studies (GWASs) have linked single-nucleotide polymorphisms (SNPs) to gastroduodenal ulcer disease,^23,24^ gastric premalignant lesions,^25–29^ and gastric cancer.^24,27–39^ Interestingly, some of the associations found in those studies seem to be influenced by the presence of *H pylori* infection,^25,29–33^ suggesting that genomic variants might be involved in *H pylori* colonization as well. The first and largest GWAS on *H pylori* combined data of Dutch and German population-based cohorts in a meta-analysis of anti–*H pylori* IgG titers by means of a dichotomic study design that compared the 25% of individuals exhibiting the highest anti–*H pylori* IgG titers vs the remaining 75%.^40^ Two loci, the Toll-like receptor (*TLR1/6/10*) locus on *4p14* (lead SNP rs10004195) and the Fc gamma receptor 2A (*FCGR2A*) locus on *1q23.3* (lead SNP rs368433), were identified to be associated with increased anti–*H pylori* IgG titers.^40^ A GWAS among Finnish male smokers (n = 1402) confirmed the lead SNP rs10004195 to be associated with the height of IgG titer rather than a seropositive status itself.^41^ However, no further replication of these findings have been reported so far, and in contrast to those European studies, no genome-wide significant associations of *H pylori* serology with any loci were found in a Mexican-American population (n = 1931).^42^ Because the main findings of the first GWAS study have not been uniformly confirmed,^40^ the present study aimed to update the original meta-analysis with the use of a larger sample size and to investigate the functional relevance of variation at the *TLR1/6/10* locus in *H pylori* colonization.

## Materials and Methods

### Study Cohorts

The discovery GWAS was conducted in subjects of European ancestry from population-based cohorts in Europe and the United States with low gastric cancer incidence to re-investigate the previous association between *TLR1/6/10* and anti–*H pylori* IgG levels, and to explore the possibility of new genetic associations. A total of 7 cohorts were included and consisted of 15,685 participants (Supplementary Table S1). The replication was conducted in 2 independent European cohorts with a total of 9676 participants with GWAS or de novo genotyping data. In all cohorts, serologic measurements of anti–*H pylori* IgG were performed by means of either commercial or customized enzyme-linked immunosorbent assay (ELISA) (Supplementary Table S1). As in the initial study, the 25% of subjects with the highest anti–*H pylori* IgG values were compared with the remaining 75% in a dichotomous study design.^40^ Informed consents for participation were obtained for all study subjects, and approvals were given by the institutional review boards. More details concerning individual cohorts are described in the Supplementary Methods.

### Discovery

Genome-wide genotyping, imputation to 1kgP1v3, and genome-wide association analyses were conducted separately by the discovery cohorts (Supplementary Methods). EasyQC using standard settings was applied for quality control of individual cohort summary statistics.^43^ The inverse-variance weighted fixed-effects model approach was used for meta-analysis with the use of METAL.^44^ A quantile-quantile plot of observed compared with expected —log_10_ (*P* value) was computed to investigate genome-wide distribution of *P* values, and a Manhattan plot to illustrate genome-wide *P* values. Genome-wide significance was set at a threshold with *P* value <5.0 × 10^−8^. A regional plot was generated to show the genomic regions within 100 kb of top hits. In addition, a random-effects model was conducted to explore the association between the *TRL* locus (rs12233670) with *H pylori* in more detail.

### Replication

Eight top SNPs with the lowest association *P* values from the discovery phase were selected for replication, particularly rs12233670 within the *TLR1/6/10* locus. The ESTHER (Epidemiological Investigations on Chances of Preventing Recognizing Early and Optimally Treating Chronic Diseases in an Elderly Population) cohort achieved in silico replication of 7 out of 8 SNPs (excluding rs147174426), and the Latvian cohort performed de novo genotyping for 4 individual SNPs (rs12233670, rs147174426, rs6107461, rs147900026) (Supplementary Methods). Replication was considered successful with *P* value <0.05 for individual cohorts and *P* value <5.0 × 10^−8^ for the combined analysis.

### Longitudinal Analysis of Serologic Data From H pylori–Positive Subsets

To determine whether the timing of anti–*H pylori* IgG testing may influence serologic outcomes relevant for genetic association studies, 2 different serologic data subsets were analyzed. The first subset consisted of RS (Rotterdam Study) participants with pharmacy records of *H pylori* eradication treatment before serology (n = 132), allowing analysis of anti–*H pylori* IgG titers in relation to time following eradication. Anti–*H pylori* antibodies were measured with the use of the Pyloriset EIA-G III (Orion Diagnostica, Espoo, Finland). The second subset consisted of patients from an ongoing prospective study aimed at the surveillance of atrophic gastritis, intestinal metaplasia, and dysplasia in the Netherlands and Norway.^45^ Anti–*H pylori* antibodies were measured as part of the GastroPanel test (Biohit, Helsinki, Finland) using serum samples collected during clinical follow-up. Patients with elevated anti–*H pylori* IgG levels (>30 enzyme immunoassay units) at baseline in addition to consecutive serum measurements (n = 107) were included to explore fluctuation of the titers over time. All subjects with positive histopathologic/urea breath test findings for *H pylori* received eradication therapy with efficacy verified by means of fecal antigen testing.

### Restriction Fragment Length Polymorphism Polymerase Chain Reaction Assay

Human genomic DNA was isolated from EDTA whole blood with the use of the Kleargene Blood DNA isolation kit (LGC, Teddington, UK) to determine the genotype of subjects included in our functional assays. A restriction fragment length polymorphism polymerase chain reaction (PCR) assay could not be designed for rs10004195, but *TLR* variant rs28393318 is in complete linkage disequilibrium (LD) (*r*^2^ = 1 among Utah residents from north and west Europe [CEUs]) and was therefore used as proxy (Supplementary Table S2). For genotyping of rs28393318, 35 cycles of PCR amplification were performed with custom-designed primers (forward: 5′-TAGCTCAGTGTAGGTGGTCT-3′; reverse: 5′-ATGATTAGT-GACCTTGGGGC-3′) at an annealing temperature of 53^◦^C. PCR products were confirmed on 2% Tris-borate-EDTA agarose gel and 10 *μ*L of amplicons were subjected to 5 international units of Hin1II restriction enzyme (Thermo Fisher, Waltham, MA) for 2.5 hours at 37^◦^C. After 20 minutes of enzyme inactivation at 80^◦^C, digestion products were visualized on agarose gel, showing 1 band of 433 base pairs (bp) for genotype GG, 2 bands of 311 and 122 bp for AA, and 3 bands for GA.

### Functional Analysis

#### Flow cytometry.

To investigate whether genetic variation at the *TLR* locus on *4p14* affects the expression of the receptor at the cell surface, the presence of TLR1, TLR6, and TLR10 was measured with the use of flow cytometry. Whole-blood samples from non–*H pylori*–infected individuals without significant comorbidities (n = 26), taken after informed consent, were treated with eBioscience 1-step Fix/Lyse Solution (Thermo Fisher) to lyse red blood cells. Monocytes and polymorphonuclear cells (PMNs) were incubated with antibodies specific for CD14 (APC-cy7, cat. no. A15453), CD66B (APC, cat. no. 17–0666-42), and TLR1 (PE, cat. no. 12–9911-42) (all from Thermo Fisher) or mouse IgG1*κ* isotype control (PE, cat. no. 554121; BD Biosciences, Franklin Lakes, NJ) for 15 minutes on ice. Because the genes encoding TLR6 (PE, cat. no. MA5–16177) and TLR10 (PE, cat. no. 12–2909-42) reside within the same genetic locus as rs28393318, the surface expression of those proteins was also measured. Flow cytometry was performed on the MACSQuant Flow Cytometer (Miltenyi Biotec, Gladbach, Germany) and analysis conducted with the use of FlowJo v10 (BD Biosciences). Monocytes and PMNs were identified on the basis of the forward/sideward scatter and further gating on CD14 and CD66b, respectively. TLR positivity was measured with gating based on the isotype control of the same sample.

#### Reverse-transcription quantitative PCR analysis of TLR1 transcript levels.

To explore whether differences in TLR1 surface expression among genotypes were attributable to variation in mRNA expression, quantitative PCR (qPCR) analysis was conducted. Total RNA was isolated from peripheral blood mononuclear cells (PBMCs) of non–*H pylori*–infected individuals without significant comorbidities (n = 22) by means of the column-based NucleoSpin RNA kit (Macherey-Nagel & Co, Düren, Germany) and reverse transcribed into complementary DNA (cDNA) with the use of PrimeScript RT (Takara, Kusastsu, Shiga, Japan). A qPCR assay of 40 cycles was performed on the StepOnePlus Real-Time PCR system (Thermo Fisher) using SYBR Select Master Mix (Thermo Fisher) and custom-designed *TLR1* gene primers (forward: 5′-TGCCAAATGGAACAGACAAGCAG-3′; reverse: 5′-ACA-GATTCCTTTTGTAGGGGTGCC-3′) and *RP2* housekeeping gene primers (forward: 5′-AAGCTGAGGATGCTCAAAGG-3′; reverse: 5′-CCCATTAAACTCCAAGGCAA-3′). The annealing temperature was 61^◦^C for both primer sets. The delta-delta cycle threshold (ΔΔCt) method was applied for data analysis.

#### ELISA for cytokine analysis on TLR1 stimulation.

To study TLR1 signaling in more detail, PBMCs from non–*H pylori*–infected individuals (n = 22) were isolated from heparinized blood as described previously.^46^ In brief, phosphate-buffered saline solution (PBS)–diluted blood was layered onto Ficoll (Amersham, Uppsala, Sweden) and PBMCs harvested after centrifugation, washed in PBS, and plated in Roswell Park Memorial Institute medium (Lonza, Basel, Switzerland) containing 10% fetal bovine serum and penicillin/streptomycin (Lonza). Two million PBMCs were seeded in 12-well plates in a total volume of 2 mL. After 24 hours of incubation, wells were washed and PBMCs stimulated with 1 million colony-forming units of heat-killed *H pylori* (strain ATCC-43504 [cagA^+^, vacA^(s1/m1)^, iceA^+^, babA2^+^]; Manassas, VA) grown on Trypticase Soy Agar (Oxoid, Hampshire, UK) supplemented with 5% defibrinated sheep blood (VWR, Radnor, PA) and DENT selective medium (Oxoid). Other stimuli used were TLR1 inhibitor CU-CPT-22 (5 *μ*mol/L; Tocris Bioscience, Bristol, UK) and TLR1 agonist Pam3Cys4 (300 ng/mL; InvivoGen, San Diego, CA).^47^ Supernates were harvested after 8 hours of stimulation for ELISA experiments unless otherwise specified to measure tumor necrosis factor (TNF) *α*, interleukin-8 (IL8), and IL10 (eBioscience, San Diego, CA) as described previously.^48^ All samples were tested in duplicate.

### Statistical Analysis of Serologic and Functional Data

Statistical differences among 3 groups were determined by means of 1-way analysis of variance or Kruskal-Wallis tests for unpaired data and repeated-measures analysis of variance or Friedman tests for paired data and was followed by post hoc analysis for selected pairs with adjustment for multiple testing. The 2-sample *t* test or Mann-Whitney test were applied to compare 2 groups with unpaired data. GraphPad Prism software version 5.01 (GraphPad Software, San Diego, CA) was used for calculations and graphic representation.

## Results

### Genomic Variants Associated With Anti–H pylori IgG Titers in an Updated GWAS

**Figure 1.**
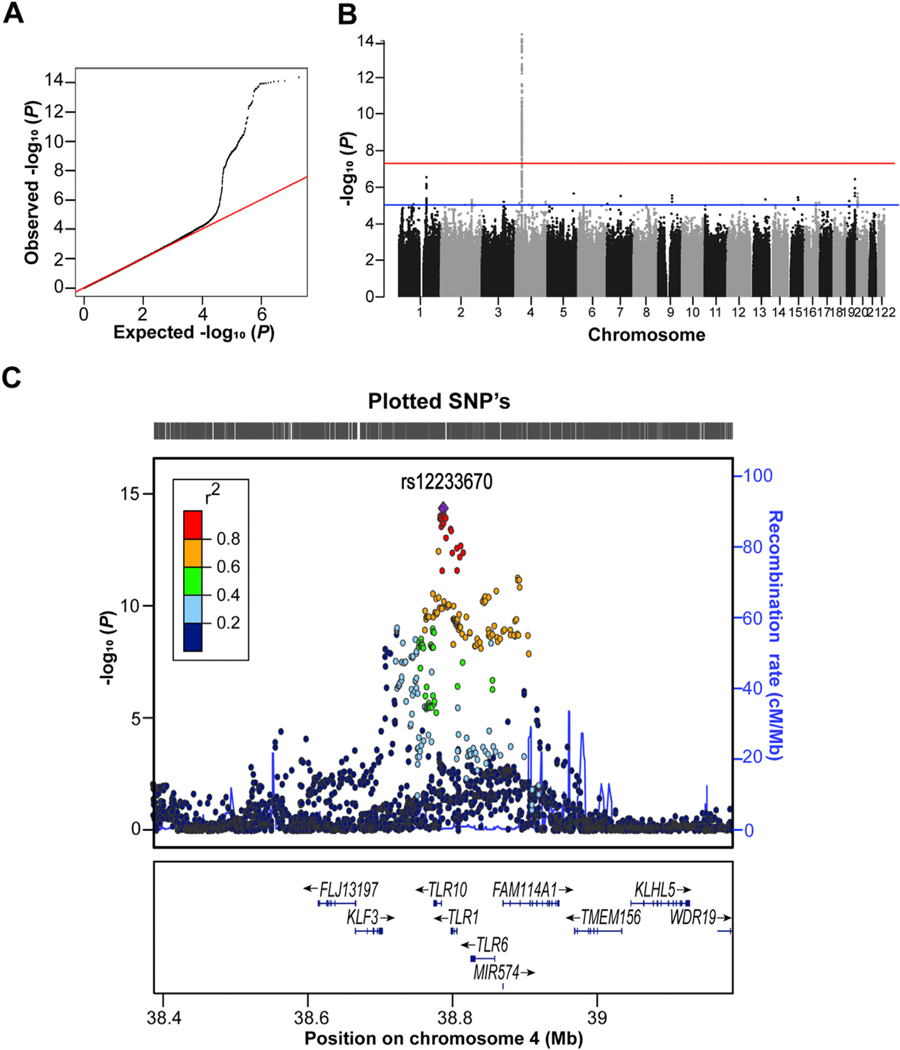
Results of genome-wide association meta-analysis for anti–*Helicobacter pylori* IgG titer. (*A*) Quantile-quantile plot with all single-nucleotide polymorphisms (SNPs) displayed as *black dots* and the r*ed line* corresponding to the null hypothesis of no true association. (*B*) Manhattan plot of the genome wide association meta-analysis with the chromosome position on the *x-*axis and —log_10_ *P* values on the *y-*axis. Each *dot* indicates an SNP, the *blue line* marks the threshold of *P* = 1.0 × 10^−5^, and the *red line* represents the genome-wide significant threshold of *P* = 5.0 × 10^−8^. (*C*) Regional plot showing locus *4p14* with rs12233670 and other flanking region markers. The *P* values of SNPs associated with *H pylori* are plotted against their chromosome position on the *x*-axis. Each SNP is represented by a *colored dot* indicating their correlation (linkage disequilibrium) with the top-ranked SNP (*purple diamond*). The *y*-axis at the right represents the recombination rates. The bottom part depicts the annotated genes at the locus and their transcriptional direction.

We performed a GWAS meta-analysis based on 7 independent European epidemiologic cohorts with the use of the fixed-effect model. The quantile-quantile plot showed a clear deviation from the null-distribution at the tail (Figure 1A). A genome-wide significant association for the *TLR1/6/10* locus on chromosome *4p14* was found with top SNP rs12233670 carrying the lowest *P* value (*β* = −0.267 ± SE 0.034 for minor allele T; *P* = 4.42 × 10^−15^; minor allele frequency = 25%) (Figure 1B and C), albeit with statistical heterogeneity (Table 1). The association between top SNP rs12233670 and anti–*H pylori* IgG titers was not significant in either ESTHER (*β* = 0.041 ± SE 0.050 for the minor allele; *P* = 0.41) or LATVIA (*β* = 0.017 ± SE 0.079 for the minor allele; *P* = 0.83) cohorts, resulting in a failed replication (*β* = 0.034 ± SE 0.042 for minor allele; *P* = 0.42) (Table 1). Consequently, the level of genome-wide significance decreased in the combined analysis (*β* = −0.149 ± SE 0.027 for the minor allele; *P* = 1.97 × 10^−8^) (Table 1). Seven other promising SNPs were identified but did not reach genome-wide significance, including the *FCGR* locus (1q23.3; top-ranked SNP rs147174426; *β* = 0.480 ± SE 0.094 for major allele A; *P* = 2.89 × 10^−7^; minor allele frequency = 7%). Similar results were obtained with the use of a sensitivity model including adult participants only (data not shown). None of these selected SNPs reached genome-wide significance in the combined analysis with discovery and replication cohorts (Supplementary Table S3).

With high inter-study heterogeneity observed for rs12233670 in the discovery, a random-effects model was applied for this particular SNP. The *P* value was no longer genome-wide significant (*P* = 0.0056), but the effect estimate was similar with a odds ratio of 1.26 (95% CI 1.07–1.48) instead of 1.3 (95% CI 1.22–1.39) obtained with the fixed-effect model.

### Anti–H pylori Antibody Decay in H pylori–Infected Subjects

**Figure 2.**
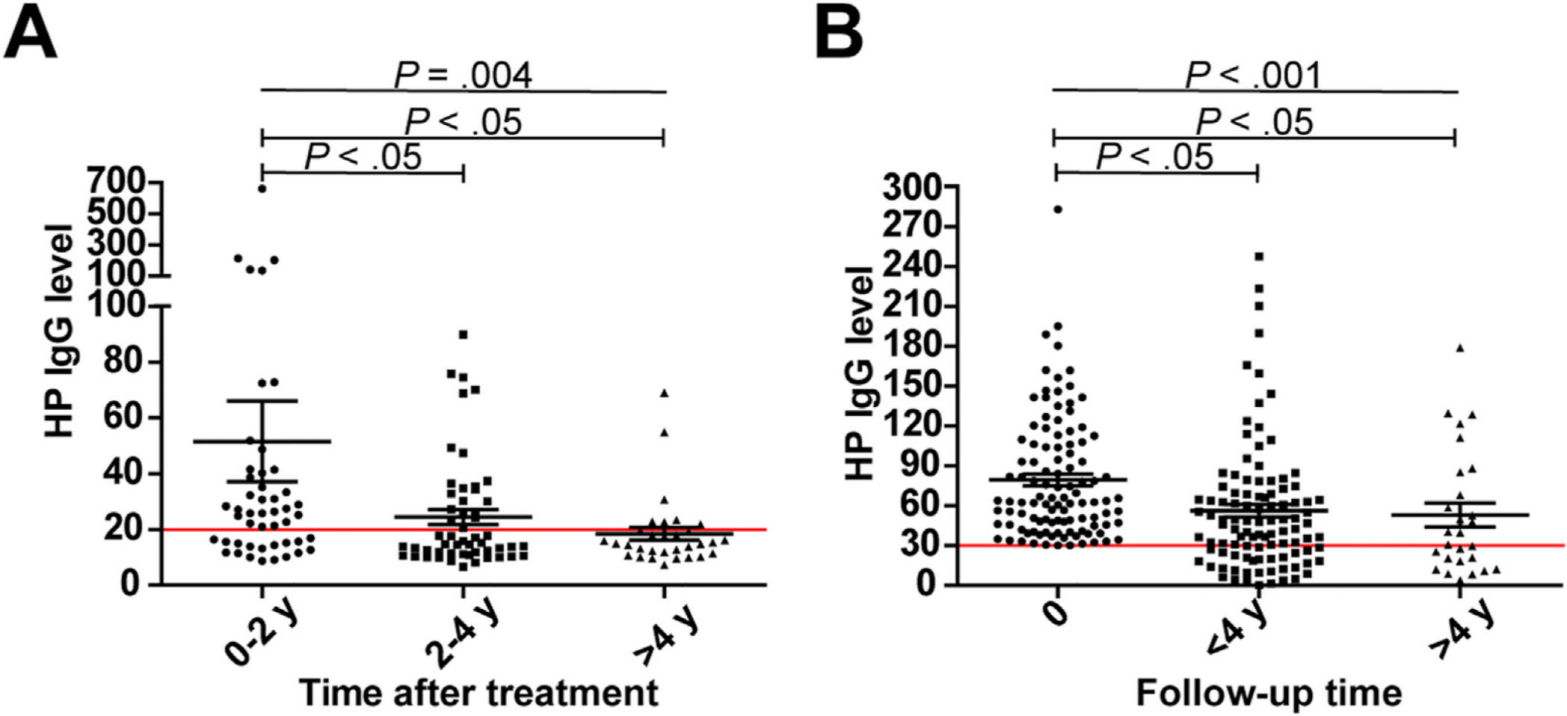
Anti–*Helicobacter pylori* (HP) IgG levels over time in 2 subsets. (*A*) IgG levels of Rotterdam Study subjects (n = 132) who received eradication therapy before serologic testing. The measurements of subjects are divided into 3 groups based on the time between treatment and *H pylori* serology: 0–2 (n = 48), 2–4 (n = 53), and >4 (n = 31) years. (*B*) IgG levels of *H pylori*–positive patients with premalignant gastric lesions (n = 107) at baseline (time point 0 with IgG titers >30 enzyme immunoassay units) and during serologic follow-up at <4 (n = 104) and >4 (n = 25) years. In both plots, the mean ± SEM and the manufacturer’s test cutoff (*red horizontal line*) are shown.

We considered that antibody decay and timing of sampling for *H pylori* serology may contribute to cohort heterogeneity. To investigate the serologic course of *H pylori*–infected subjects, anti–*H pylori* IgG data were studied in 2 settings. In a subset of RS participants that received *H pylori* eradication treatment at some point before the measurement of IgG antibodies (n = 132), titers were significantly higher in individuals tested within 0–2 years (n = 48) after receiving eradication therapy than in those tested 2–4 (n = 53) or >4 (n = 31) years (*P* = 0.004) after treatment (Figure 2A). When analyzing sequential anti–*H pylori* IgG titers from patients with gastric premalignant lesions with positive *H pylori* serology (n = 107), a significant decline between baseline measurement (time point 0) and retesting at <4 (n = 104) or >4 (n = 25) years of medical follow-up (*P* < 0.001) was seen (Figure 2B). Together, these data indicate that anti–*H pylori* antibody decay occurs within 2 years after treatment or clearance of *H pylori*.

### Higher TLR1 Surface Protein but Not Intracellular mRNA Expression Levels in Leukocytes of G Allele Carriers of rs28393318

**Figure 3.**
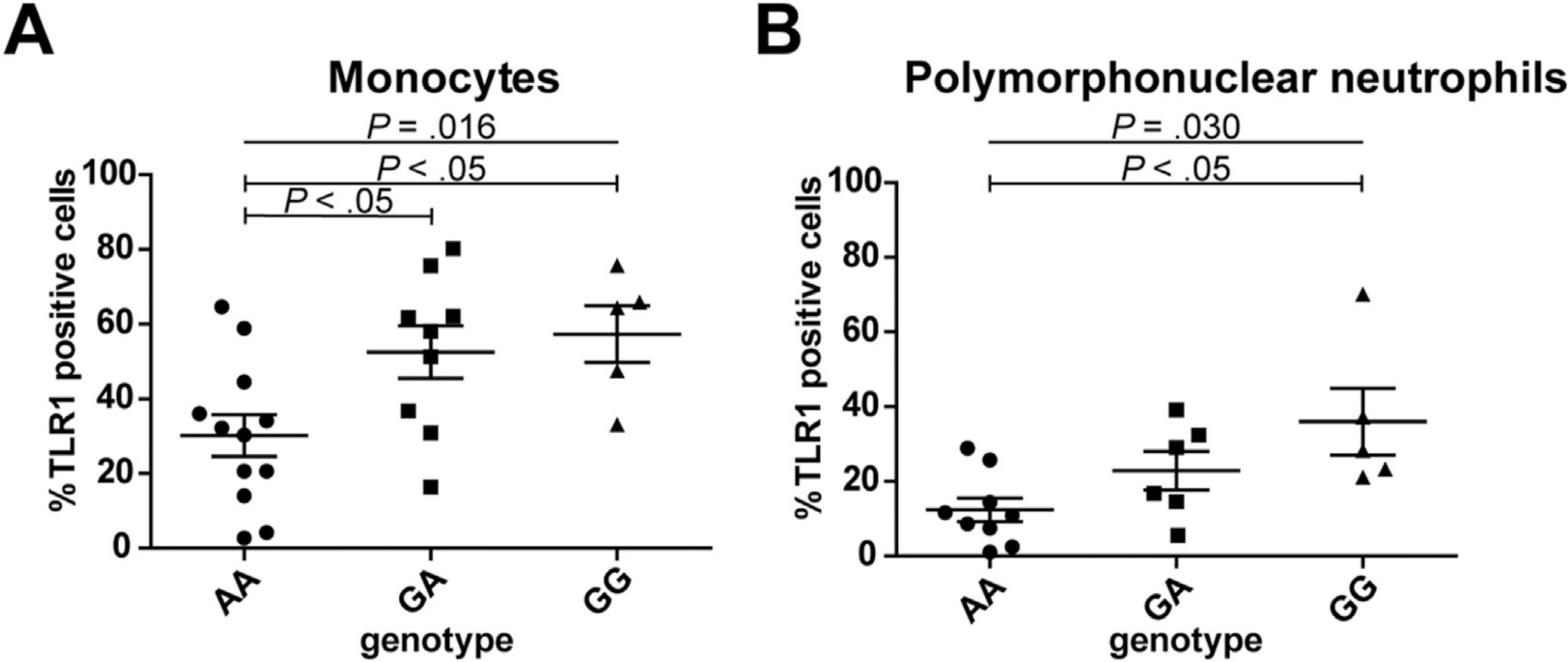
Measurement of Toll-like receptor 1 (TLR1)–positive cells by flow cytometry. Dot plots illustrating the percentage of TLR1 positive cells in healthy subjects genotyped for rs28393318. The mean ± SEM and statistical significance among 3 genotypes are shown. (*A*) TLR1 positivity of monocytes in AA (n = 12), GA (n = 9), and GG (n = 5) carriers. (*B*) TLR1 positivity of polymorphonuclear neutrophils in AA (n = 9), GA (n = 6), and GG (n = 5) carriers.

To investigate potential functional consequences of variation at the *4q14* locus, expression of the TLR-encoding genes within this locus was investigated in healthy subjects (n = 26) genotyped for rs28393318. A significant difference in the percentage of TLR1-positive monocytes (*P* = 0.016) and PMNs (*P* = 0.030) was observed between AA, GA, and GG genotype carriers (Figure 3A and B). Post hoc analysis revealed significantly higher TLR1 surface expression on monocytes for carriers of the minor rs28393318 allele (G) and on PMNs in subjects homozygous for the G allele compared with A allele carriers. Variation at rs28393318 did not influence TLR6 and TLR10 surface expression on either monocytes or PMNs (Supplementary Figure S1).

Unlike previous RNA sequencing–based findings of reduced *TLR1* mRNA levels for minor rs10004195 A allele carriers,^40^ our reverse-transcription qPCR findings showed no differences in *TLR1* transcript levels in PBMCs between healthy subjects (n = 22) with different genotypes of rs28393318 (Supplementary Figure S2), which is in line with previous reports demonstrating no differences in mRNA and total cellular TLR1 protein levels among other TLR1 variants (in high LD with rs28393318 and rs10004195 among CEUs) tested.^49–52^

### TLR1 rs28393318 Affects Immune Cell Cytokine Production

**Figure 4.**
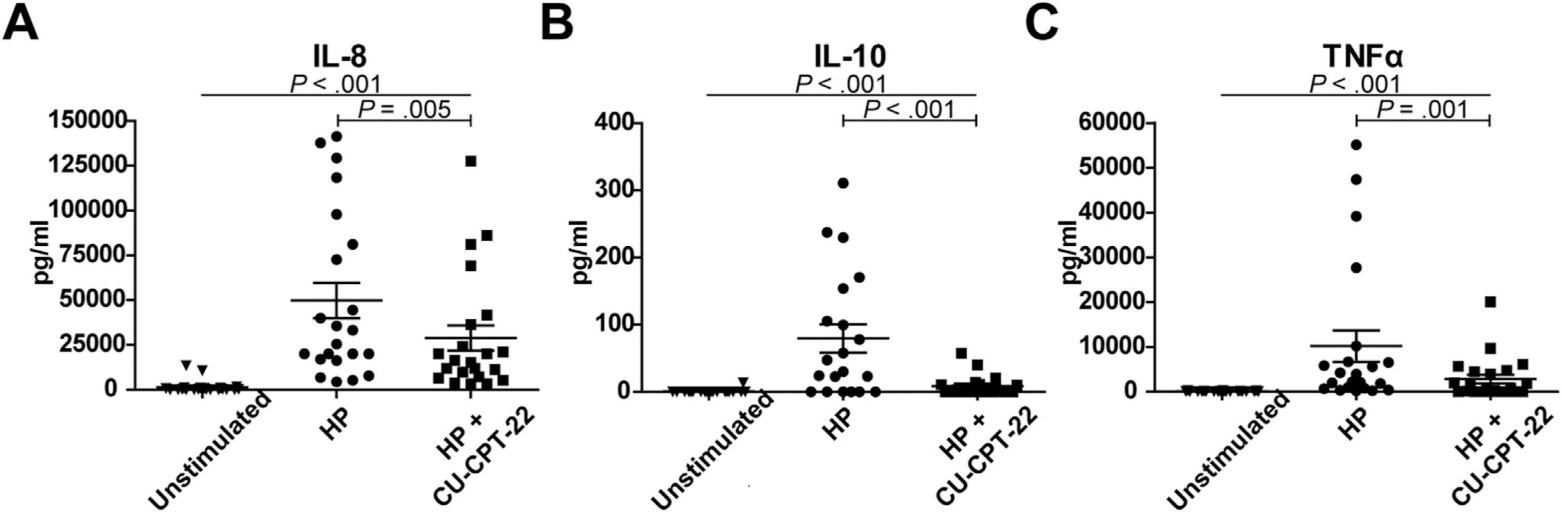
Cytokine production by peripheral blood mononuclear cells (PBMCs) on stimulation with *Helicobacter pylori*. (*A*) Interleukin-8 (IL8), (*B*) interleukin-10 (IL10), and (*C*) tumor necrosis factor α (TNFα) levels in PBMCs of healthy subjects (n = 22). Cytokine levels were measured at baseline without stimulation and after *H pylori* exposure in the absence and presence of TLR1 inhibitor CU-CPT-22.

To first confirm TLR1 involvement in *H pylori* pathogenesis, PBMCs from healthy subjects (n = 22) were treated with *H pylori* in the presence of selective TLR1 inhibitor CU-CPT-22 or vehicle control. *H pylori* significantly stimulated IL8, IL10, and TNFα production (all *P* < 0.001), which was significantly but not fully abrogated on treatment of cells with CU-CPT-22 (*P* = 0.005 for IL8; *P* < 0.001 for IL10; *P* = 0.001 for TNFα) (Figure 4). These findings suggest that *H pylori*–related cytokine signaling is partly mediated via TLR1.

**Figure 5.**
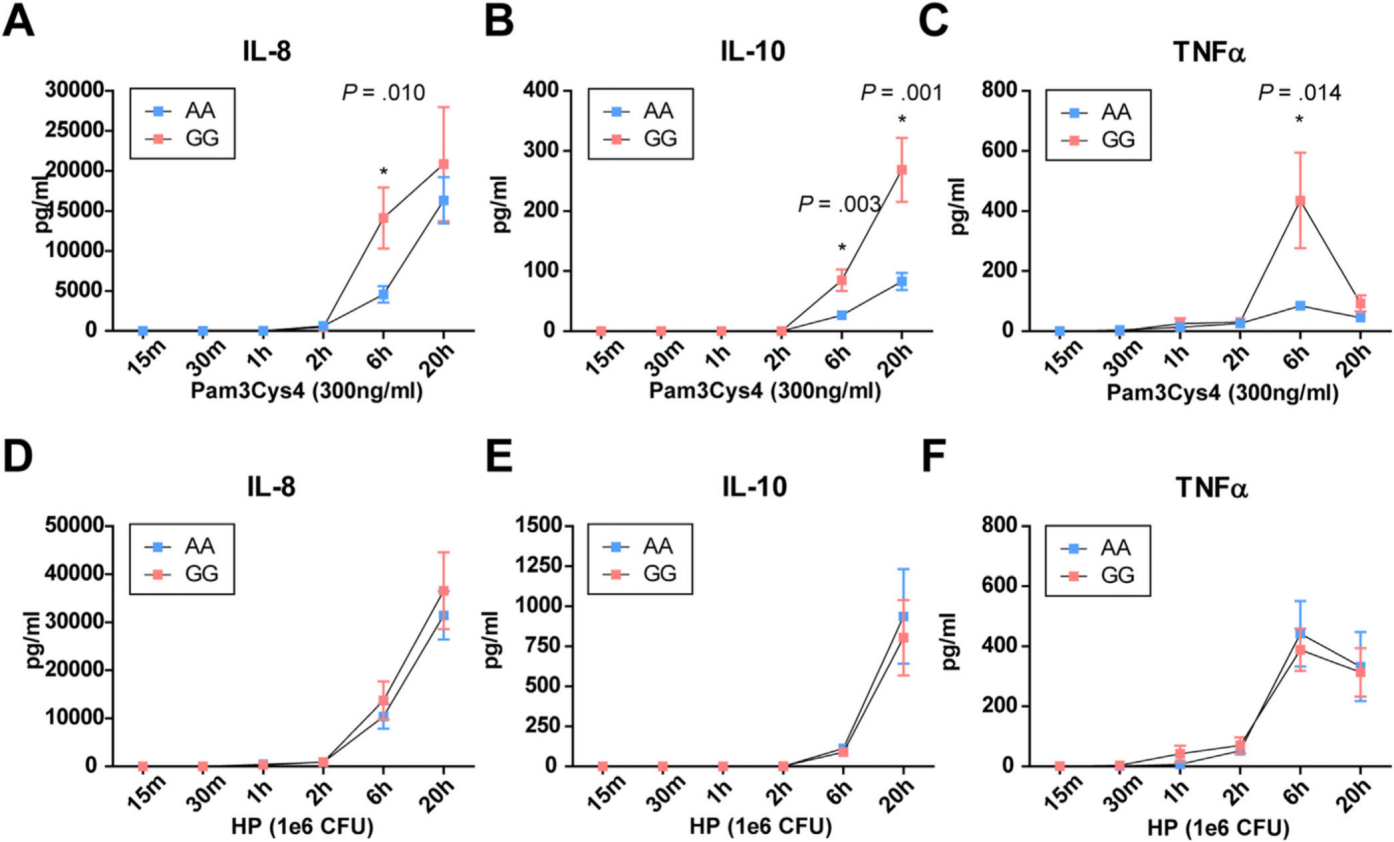
Time course of cytokine production by peripheral blood mononuclear cells (PBMCs) on stimulation with Toll-like receptor 1 (TLR1) ligand Pam3Cys4 or *Helicobacter pylori* in AA and GG carriers of rs28393318. (*A, D*) Interleukin-8 (IL8), (*B, E*) IL10, and (*C, F*) tumor necrosis factor α (TNFα) levels measured at different time points after stimulation with (*A*–*C*) TLR1 agonist Pam3Cys4 or (*D*–*F*) *H pylori* (HP). The results are stratified for AA (n = 12) and GG (n = 8) carriers of rs28393318. The mean ± SEM and statistical significance between genotypes are shown. CFU, colony-forming unit.

We next explored TLR1-mediated differences in cytokine production between healthy subjects with rs28393318 genotypes AA (n = 12) and GG (n = 8). PBMCs were stimulated with the specific TLR1 agonist Pam3Cys4 or with *H pylori*, and cytokine levels were measured at different time points afterward (0.25, 0.5, 1, 2, 6, and 20 hours). Pam3Cys4 stimulation resulted in higher IL8 (*P* = 0.010), IL10 (*P* = 0.003), and TNFα (*P* = 0.014) production at 6 hours as well as higher IL10 levels at 20 hours (*P* = 0.001) in GG carriers compared with AA carriers. Cytokine production on *H pylori* treatment was considerably higher than on Pam3Cys4 stimulation, but no differences among genotypes were observed (Figure 5).

## Discussion

This study aimed to better understand the genome-wide association between the *TLR1/6/10* locus and *H pylori*. We extended the original work of Mayerle et al^40^ by the inclusion of an additional 4747 subjects of European ancestry in an updated GWAS meta-analysis. An association between anti–*H pylori* IgG titers and the *TLR1/6/10* locus with top SNP rs12233670 was demonstrated in the discovery phase using the fixed-effect model, but replication proved to be challenging. Significant heterogeneity for our top association was observed across cohorts, with the SHIP-TREND and both replication cohorts showing association in the opposite direction. The interpretation of these findings remains complex but might be partially explained by methodologic differences that are inherent in the inclusion of longitudinally population cohorts, including time of recruitment (eg, SHIP vs SHIP-TREND) and use of nonuniform serologic assays. The concession of accounting for false positive assignment of cases by using the 25% highest vs 75% lowest IgG distribution might be another explanation. The allocation of truly *H pylori*–infected subjects into the control group could have possibly limited the detection and replication of promising SNPs, particularly for high-endemic regions such as Latvia.^53^ On the other hand, using a dichotomous cutoff based on IgG titers rather than test-defined *H pylori* positivity precludes bias introduced by the use of different tests with varying sensitivity and specificity across the different cohorts. Heterogeneity in our study may also have been introduced through antibody decay. Studying 2 different serologic datasets, we demonstrated that anti–*H pylori* IgG antibody decay over time occurs relatively quickly, as was previously observed on *H pylori* eradication treatment.^54^ Knowing that this process takes place, it is imperative to know the time of collection in relation to *H pylori* infection. Different rates of spontaneous clearance, re-infection, and *H pylori* eradication might have contributed to study heterogeneity, but this information was unfortunately not routinely collected in addition to data regarding *H pylori*–related disease status (eg, gastric cancer). Finally, various *H pylori* strains with varying virulence may interact differently with their human host, influencing the clinical outcome.^55^

Despite technical challenges preventing a clear replication, several studies do point toward a role of the *TLR1/6/10* locus in the interaction between *H pylori* and its human host.^40,41^ With the data of the discovery phase showing an association with anti–*H pylori* IgG titers in the same direction as the original report, the relevance of the *TLR1/6/10* locus in relation to *H pylori* pathology was further indicated. Our functional experiments demonstrate that variation at the *TLR* locus indeed has functional implications, as shown by a higher TLR1 surface expression and higher cytokine production in minor allele G carriers of rs28393318 (which is in complete LD with our top SNP rs12233670 and rs10004195 among CEUs). This might be explained by 2 non-synonymous *TLR1* variants affecting intracellular–to–cell surface trafficking (rs5743618; *r*^2^ = 0.86 with rs28393318 among CEUs) and transportation of the receptor to the cell membrane (rs4833095; *r*^2^ = 0.97 with rs28393318 among CEUs).^49–52,56,57^ Minor allele carriers of these *TLR1* genetic variants displayed higher cytokine responses on targeted TLR1 stimulation (ie, with Pam3Cys4), which was attributed to increased TLR1 surface expression rather than to changes in total protein or mRNA levels measured in cells,^49–52^ which is in line with our findings. Although *H pylori* mediates IL8, IL10, and TNFα production at least partially via TLR1 signaling in PBMCs, *H pylori*–mediated cytokine production was not affected by rs28393318 status of carriers. It is likely that rs28393318 variation effects are masked by other components of the host immune system triggered by this highly virulent *H pylori* strain.^58^ Similarly, the effect of rs28393318 variation on serologic titers induced by *H pylori* infection may be masked by additional *H pylori*–induced host-specific immune responses.

This study has 2 major limitations that need to be addressed. First, the identification of new genetic variants and replication of promising candidates for anti–*H pylori* IgG titers may have been hampered by the chosen definition of seropositivity for *H pylori* antibodies. Future studies might have to reconsider the phenotype definition, because the interpretation of *H pylori* serologic determination is not straightforward. Anti–*H pylori* IgG levels are more likely to represent a combination of the host’s ability to mount an immunologic response to infection as well as antibody clearance than actual *H pylori* incidence. With time from *H pylori* eradication therapy to serum collection influencing IgG antibody titers, it would be of value to collect those data in future studies. It should also be noted that *H pylori* infection involves an interplay of factors (host, bacterial, and environmental). Many genetic variants have been identified for *H pylori*–related conditions, such as ulcerative and (pre)malignant gastric lesions in different ethnic populations,^23–25,27–32,38^ and therefore it seems plausible that other genetic variants are relevant in *H pylori* pathogenesis besides the *TLR* locus. Second, we tested a selective cytokine panel in our functional analysis as a proof of concept. To better understand the immune response with regard to *H pylori* susceptibility, future experiments with different *H pylori* strains in different ethnic populations would be of interest.

In summary, the previously observed association between the *TLR1/6/10* locus and anti–*H pylori* antibody titers was not uniformly confirmed across cohorts in this study. The interpretation of *H pylori* serology is complex and subject to alterations in response to therapy and over time. While variation at the *TLR1/6/10* locus regulates surface expression and cytokine production on stimulation, further efforts are required to better understand the clinical relevance of *TLR* variants and other loci in their complex interaction with *H pylori*.

## Supplementary Material

Supplementary Material

## Figures and Tables

**Table 1.** Summary of Single-Nucleotide Polymorphism (SNP) at the *TLR1/6/10* Locus in Discovery, Replication, and Combined Meta-analysis

ID	Chr	Position	Gene^a^	A1/2 ^b^	EAF	Beta^c^	SE	*P* value	*I* ^2^

					Discovery				
rs12233670	4	38787216	*TLR10*	T/C	0.25	−0.267	0.034	4.42 × 10^−15^	79.8
					Replication				
					0.20	0.034	0.042	0.417	0.0
					Combined				
					Beta^c^	SE	Dir^d^	*P* value	*I* ^2^
					−0.149	0.027	−++	1.97 × 10^−8^	30.9

Chr, chromosome; EAF, effect allele frequency; *I*^2^, measure of heterogeneity.

a Gene nearest to the SNP.

b A^1^/_2_, effect allele 1 and other allele 2.

c Effect size is relative to the allele 1.

d Direction of beta of, respectively, the discovery, ESTHER, and LATVIA cohorts: positive (+) or negative (−).

## WHAT YOU NEED TO KNOW

### BACKGROUND

The identification of a genome-wide association between the Toll-like receptor (*TLR*) locus and anti–*Helicobacter pylori* IgG titers presented a major breakthrough, but this link is complex and not fully understood.

### NEW FINDINGS

Genetic variation at the *TLR1/6/10* locus confers immunologic cellular consequences, but high heterogeneity, cohort differences, and antibody decay likely prevent replication of previous associations with anti–*H pylori* IgG.

### LIMITATIONS

Interpretation of study heterogeneity remains difficult owing to the inclusion of different longitudinal population-based cohorts for this largest dichotomous genome-wide association study for anti–*H pylori* IgG titers to date.

### IMPACT

A role for the *TLR1/6/10* locus in host–*H pylori* interaction is likely, but alternative definitions for *H pylori* positivity may be required.

## Abbreviations used in this paper:

CEUs: Utah residents from north and west Europe
ELISA: enzyme-linked immunosorbent assay
FCGR: Fc gamma receptor
GWAS: genome-wide association study
IL: interleukin
LD: linkage disequilibrium
PBMCs: peripheral blood mononuclear cells
PMNs: polymorphonuclear cells
PCR: polymerase chain reaction
SNP: single-nucleotide polymorphism
TLR: Toll-like receptor
TNF: tumor necrosis factor
