# Supplementary material for: Toll-Like Receptor 1 Locus Re-examined in a Genome-Wide Association Study Update on Anti–Helicobacter pylori IgG Titers
Source: Gastroenterology. Author manuscript; Available in PMC 2025 Jan 15. (PMC11734630; doi:10.1053/j.gastro.2022.01.011)
Supplement: Supplementary Material [file NIHMS2041402-supplement-Supplementary_Material.pdf]

Toll-like receptor 1 locus re-examined in a genome-wide association study update on anti-*Helicobacter pylori* IgG titers

Appendix

Supplementary Methods...
1. Discovery cohorts ... 2
2. Replication cohorts ... 3
3. Ethics ... 4
4. Stage 1: discovery ... 4
5. Stage 2: replication ... 4

Supplementary Tables ...
Supplementary Table S1 ... 5
Supplementary Table S2 ... 6
Supplementary Table S3 ... 7

Supplementary Figures ...
Supplementary Figure S1 ... 8
Supplementary Figure S2 ... 9

Acknowledgments ... 10

Supplementary references ... 11

Supplementary methods

1. Discovery cohorts

The genome-wide association study (GWAS) of Mayerle *et al.* was comprised of data from the Rotterdam Study (RS) and the Study of Health in Pomerania (SHIP) with a total of 10,938 participants.[1] For our current meta-analysis, a total of 15,685 participants of European ancestry with GWAS data and *H. pylori* serology measurements were included from RS-I (n=4771), RS-II (n=2112), SHIP (n=3830), SHIP-TREND (n=983), Framingham Heart Study (FHS) (n=3141), Multi-Ethnic Study of Atherosclerosis (MESA) (n=447) and Generation R (GenR) study (n=401) cohorts.

<u>RS-I and RS-II</u>

The RS study is an ongoing prospective Dutch study in which participants of the well-defined district Ommoord in Rotterdam were enrolled in sequential cohorts over time to investigate a variety of diseases commonly in the elderly.[2-4] Baseline recruitment of the first three cohorts (RS-I, RS-II, RS-III) dated from respectively 1990-1993 (n=7983), 2000-2001 (n=3011) and 2006-2008 (n=3932), and was extended with a fourth cohort that started in 2016 (RS-IV). All participants were interviewed and underwent an extensive set of examinations focusing on possible causes of invalidating diseases in the elderly. Examinations were repeated every 3-4 years in sequential examination cycles, with the emphasis on imaging and the collection of biological specimens.

<u>SHIP and SHIP-TREND</u>

SHIP is a population based study which was set up in the North-East of Germany to investigate a wide range of health-related conditions.[5, 6] The main objectives of the study were to assess the prevalence and incidence of common risk factors, subclinical disorders and clinical diseases, and to investigate the complex associations among them. It comprised of two independent cohorts including 20-79 year old participants during 1997-2001 (n=6265) and 2008-2012 (n=8016) for SHIP and SHIP-TREND, respectively. Baseline data collection were obtained from a health-related interview, a health- and risk questionnaire and a medical examination during which biological samples were taken. Further information was collected during follow-up, including morbidity and mortality data.

<u>FHS</u>

FHS is a longitudinal multigenerational study which was originally set up to investigate the epidemiology of cardiovascular diseases in the residents of Framingham, as described previously.[7-11] After completion of the baseline recruitment of the Original cohort in 1953 (n=5209), the process was repeated for the Offspring and Spouses cohort in 1971-1975 (n=5124), the Third Generation cohort in 2002-2005 (n=4095) and the Offspring Spouses cohort during 2003-2005 (n=103). While these subjects were predominantly of Western-European descent, the Omni 1 (n=507) and 2 (n=410) cohorts represent the multi-ethnic society in Framingham.[11] Examinations cycles were performed every 2-6 years and included the collection of biological samples.

MESA

MESA is large population based study in the United States designed to study factors influencing the conversion of subclinical cardiovascular disease to overt disease in four different ethnic groups (Caucasian-, African-, Hispanic-, and Asian- American).[12-14] Study subjects were 48-84 years old and free of overt signs of cardiovascular disease at baseline enrolment in 2000-2002 (n=6814) and recruited from six field centers across the United States. At baseline, extensive data was obtained from comprehensive questionnaires, the collection of biological samples and physical assessments incorporating various imaging technologies. Follow-up examinations were conducted at ~18 month intervals in MESA.

GenR Study

The GenR Study is a population based prospective cohort study from fetal life to young adulthood and was conducted in the city of Rotterdam, the Netherlands.[15-18] GenR was designed for the long-term follow-up of a prenatally recruited multi-ethnic birth cohort to identify early environmental and genetic causes and also pathways leading to normal and abnormal growth, development and health in children The first 'Generation R' cohort is still ongoing after recruitment in 2001 (n=9749) and 'Generation R *Next'* has been launched in 2017 as the second cohort of the GenR study.

2. Replication cohorts

The replication was performed in participants of European ancestry from the epidemiological investigations on chances of preventing recognizing early and optimally treating chronic diseases in an elderly population (ESTHER) study and the Latvia cohort with respectively 6112 and 3564 subjects.

ESTHER

The German ESTHER study is a population based cohort study aimed at the prevention, early detection and optimized treatment of chronic diseases.[19-21] This ongoing study in the federal State of Saarland was conducted for the longitudinal follow up of residents which were recruited at the age of 50-74 years during medical check-up in 2000-2002 (n=9940). A health check-up documentation was provided by the general practitioner and a standardized questionnaire regarding sociodemographic, lifestyle factors and medical history was collected from the study participant. Biological samples were obtained at baseline and follow-ups after 5, 8, 11, 14 and 17 years.

Latvia cohort

The Latvia cohort consists of a cross-sectional subset of adults from the National Latvian population in the period of 2008-2009 to study cardiovascular risk factors.[22, 23] A total of 4198 subjects agreed on participation, 4022 came for a visit, and 3807 completed the interview and allowed blood collection for biochemical and genetic analysis. Phenotypic data was stored in the Institute of Cardiology at the University of Latvia and genotyping data at the

Latvian Biomedical Research and Study Centre (BMC). The latter maintains the Genome Database of the Latvian Population (LDGB), a national biobank that provides biologic specimens and associated phenotypic and clinical data for genetic and biomolecular research purposes.[24]

3. Ethics

Informed consent for participation was obtained for all study subjects and approval was given by the Institutional Review Boards of the Erasmus University Medical Center Rotterdam (RS-I, RS-II, GenR, healthy controls), University Medicine Greifswald (SHIP, SHIP-TREND), Boston University Medical Center (FHS), Johns Hopkins University (MESA), Northwestern University (MESA), Wake Forest University (MESA), University of California at Los Angeles (MESA), Columbia University (MESA), University of Minnesota (MESA), Heidelberg University (ESTHER), Medical Association of Saarland (ESTHER) and the University of Latvia (Latvia cohort).

4. Stage 1: discovery

Different platforms for genome-wide genotyping were employed by discovery cohorts using standard procedures of the manufacturer. The Illumina platform was used by RS-I (HumanHap 550K (V.3) single and duo arrays), RS-II (HumanHap 550K (V.3) duo and Human 610K Quad arrays), SHIP-TREND (HumanOmni2.5-Quad array) and the GenR study (HumanHap 610K and 660K Quad arrays). The Affymetrix platform was employed by SHIP and MESA (both Genome-Wide Human SNP array 6.0) in addition to FHS (Human Mapping 500K plus 50K supplemental arrays). To aid meta-analysis, all datasets were imputed to the 1000 genome (1KG) dataset version 1v3 (with 30 million resulting SNP genotypes). Genome-wide association analyses were performed in individual cohorts with adjustment for sex, age and study specific covariates.

5. Stage 2: replication

Genotyping data of the ESTHER cohort was generated on the Illumina platform (Infinium OncoArray-500K BeadChip) and imputed with 1000 genome (1KG) dataset version 1v3. *In silico* data for replication in the ESTHER cohort was available for seven out of eight SNPs including rs12233670, rs12985060, rs6107461, rs79710468, rs138776142, rs147900026, rs3905275, but not rs147174426. Individual SNP genotyping for four SNPs was applied in the Latvian cohort using a TagMan probe-based assay (Life Technologies, Carlsbad, CA). A total of four SNPs were available for replication (rs12233670, rs147174426, rs6107461 and rs147900026). The analyses were adjusted for age, sex and study specific covariates, and the replication results were included in the combined meta-analysis.

Supplementary Table S1 Characteristics of discovery and replication cohorts

Study [a]	Country	Cohort [b]		Time (year) [c]		H. pylori IgG serology [d]				Serological subgroups [e]						
		D/R	Total	Serum collection		Test	Cut-off	Sensitivity (%)	Specificity (%)	Test cut-off				25-75% cut-off		
				From	*To*					*Status*	*n*	*Mean (SD)*	*Median (range)*	*n*	*Mean (SD)*	*Median (range)*
RS-I	The Netherlands	D	4771	1989	2004	1	≥20 U/mL	Reference [1]	Reference [1]	HP +	2826	219.5 (315.4)	102.8 (20.0 – 5587.4)	1199	441.4 (384.1)	317.6 (134.7 - 5587.4)
								97.9	58.0	HP -	1945	12.8 (2.8)	12.1 (8.0 – 19.9)	3572	32.5 (30.9)	17.9 (8.0 - 134.5)
RS-II	The Netherlands	D	2112	2000	2001	1	≥20 U/mL	Reference [1]	Reference [1]	HP +	978	183.3 (230.4)	100.7 (20.1 - 2331.4)	526	304.6 (258.0)	231.3 (88.9 - 2331.4)
								97.9	58.0	HP -	1134	12.1 (2.9)	11.2 (6.2 - 19.9)	1586	20.6 (17.1)	13.0 (6.2 - 88.9)
SHIP	Germany	D	3830	1997	2001	1	>20 U/mL	Reference [1]	Reference [1]	HP +	2269	145.4 (132.9)	95.2 (20.1 - 500)	958	268.6 (119.9)	230.8 (124.7 - 500)
								97.9	58.0	HP -	1561	12.3 (2.9)	11.3 (5 - 20)	2872	31.9 (29.3)	18.1 (5 - 124.5)
SHIP-	Germany	D	983	2008	2010	1	>20 U/mL	Reference [1]	Reference [1]	HP +	440	160.8 (152.3)	98.7 (20.1 - 500)	246	257.8 (141.2)	221.1 (78 - 500)
TREND								97.9	58.0	HP -	543	11.8 (2.8)	11 (5 - 19.8)	737	18.6 (14.4)	12.3 (5 - 77)
FHS	United States	D	3141	1991	1994	2	EV > 2.2	Reference [2]	Reference [2]	HP +	694	1.7 (0.05)	1.7 (0.5)	765	1.7 (0.0)	1.7 (0.0)
								98.4	96.4	HP -	2447	5.3 (1.4)	5.9 (4.3)	2376	5.0 (1.6)	5.7 (4.7)
MESA	United states	D	447	1999	2002	3	IV ≥1.1	Manufacturer	Manufacturer:	HP +	116	4.47 (2.41)	4.6 (1.1-10.3)	116	4.47 (2.41)	4.6 (1.1-10.3)
								94.9	90.4	HP -	331	0.35 (0.18)	0.3 (0.1-0.9)	344	0.36 (0.20)	0.30 (0.1-1.0)
GenR	The Netherlands	D	401	2006	2014	4	ODR ≥1	Reference [3]	Reference [3]	HP +	72	1.9 (1.1)	1.5 (1.1 -7.4)	100	1.5 (1.2)	1.3 (0.2 - 7.4)
								>93.0	>93.0	HP -	329	0.1 (0.06)	0.1 (0.0 - 0.2)	301	0.1 (0.05)	0.1 (0.0 - 0.2)
ESTHER	Germany	R	6112	2000	2002	5	>7.5 U	Reference [4]	Reference [4]	HP +	1755	22.3 (10.9)	20.6 (7.0 - 58.4)	1528	24.3 (10.1)	23.3 (9.52 - 58.4)
								96.0	74	HP -	4357	1.7 (1.6)	1.1 (0.0 -7.0)	4584	2.0 (2.1)	1.2 (0.0 - 9.4)
Latvia	Latvia	R	3564	2008	2009	6	>24 U/mL	Manufacturer	Manufacturer	HP +	2823	133.9 (54.3)	148.1 (24.0 - 251.1)	891	189.9 (19.6)	185.8 (164.2 - 251.1)
								<97-100	98	HP -	741	10.1 (6.1)	8.5 (1.3 - 24.0)	2673	80.1 (58.3)	79.9 (1.3 - 164.2)

[a] Study cohorts: Rotterdam Study I and II (RS-I and RS-II); Study of Health in Pomerania that consist of SHIP and SHIP-TREND; Framingham Heart Study (FHS); Multi-Ethnic Study of Atherosclerosis (MESA); Generation R study (GenR); Epidemiological investigations on chances of preventing recognizing early and optimally treating chronic diseases in an elderly population (ESTHER); Latvia cohort.

[b] Division into discovery (D) or replication (R) cohorts with number of participants with *Helicobacter pylori* serology data combined with either GWAS or genotyping data

[c] Timeframe of collection of serum samples.

[d] Enzyme-linked immunosorbent assay used to measure IgG antibodies against *Helicobacter pylori* with test cut-off according to the manufacturer, sensitivity and specificity. [1] Pyloriset EIA-G III, Orion Diagnostica, Espoo, Finland; expressed as units per milliliter (U/mL) [2] HM-CAP EIA,Enteric Products Inc., Wesbury, NY; expressed as EIA value (EV), [3] Is-H.pylori IgG EIA , Diamedix Corporation, Miami, FL; expressed as index value (IV), [4] Customized EIA; expressed as optimal density ratio (ODR), [5] H. pylori Screening EIA, Ravo Diagnostika, Freiburg, Germany, expressed as units (U), [6] recomWell Helicobacter IgG EIA Mikrogen Diagnostik, Neuried, Germany, expressed in units per milliliter (U/mL). References: [1] Hanvivatvong O *et al.*, Clin. Diagn Lab Immunol, 2014; [2] Marchildon PA *et al.*, J Clin. Microbiol, 1996; [3] Perez-Perez GI *et al.*, Ann Intern Med, 1988; [4] Brenner H *et al.*, Int. J. Cancer, 2007.

[e] Number, mean (SD) and median (range) of titers in subgroups with *Helicobacter pylori* positive (+) and negative (-) serology data using the test cut-off and the 25-75% cut-off.

Supplementary Table S2 Overview of single nucleotide polymorphisms (SNPs) of interest

SNP	Linkage disequilibrium (R^2) [a]			Location [b]		Allele [c]	Context [d]
	rs10004195	rs12233670	rs28393318	Chr	Pos	Major/minor	
rs10004195	-	1.0	1.0	4	38784724	T/A	Top-ranked SNP original report
rs12233670	1.0	-	1.0	4	38787216	C/T	Top-ranked SNP current report
rs28393318	1.0	1.0	-	4	38784267	A/G	Proxy variant for functional analysis

[a] SNPs in linkage disequilibrium in CEU
[b] Chromosome (Chr) and position (Pos) of SNP
[c] Major and minor alleles in CEU
[d] Relevance of SNP in context of current study

Supplementary Table S3 Summary of seven other single nucleotide polymorphisms (SNPs) in discovery, replication and combined meta-analysis

SNP					Discovery					Replication					Combined				
**ID**	**Chr**	**Position**	**Gene** [a]	**A½** [b]	**EAF**	**Beta** [c]	**SE**	***P*-value**	**I^2**	**EAF**	**Beta** [c]	**SE**	***P*-value**	**I^2**	**Beta** [c]	**SE**	**Dir** [d]	***P*-value**	**I^2**
rs147174426	1	161528012	*FCGR3A*	A/T	0.93	0.480	0.094	2.89×10^{-07}	23.5	0.95	-0.146	0.123	0.237	0.0	0.250	0.075	+?-	8.33×10^{-04}	16.3
rs12985060	19	47145658	*DACT3*	T/C	0.28	0.182	0.036	3.69×10^{-07}	42.2	0.19	-0.148	0.074	0.044	0.0	0.118	0.032	+-?	2.56×10^{-04}	16.2
rs6107461	20	4412588	*ADRA1D*	T/C	0.04	0.338	0.071	2.23×10^{-06}	3.5	0.03	-0.102	0.101	0.313	26.5	0.193	0.058	+--	9.09×10^{-04}	14.0
rs79710468	5	155366973	*SGCD*	A/C	0.97	-0.519	0.110	2.25×10^{-06}	0.0	0.99	-0.158	0.270	0.558	0.0	-0.468	0.102	--?	4.42×10^{-06}	1.5
rs138776142	9	82093055	*TLE4*	A/T	0.01	0.677	0.145	2.88×10^{-06}	4.1	0.01	-0.202	0.228	0.375	0.0	0.424	0.122	+-?	5.33×10^{-04}	10.6
rs147900026	7	82468050	*PCLO*	T/G	0.99	-0.756	0.162	3.10×10^{-06}	0.0	0.99	0.381	0.286	0.183	0.0	-0.480	0.141	-++	6.68×10^{-04}	12.0
rs3905275	15	60833019	*RORA*	A/C	0.82	-0.170	0.037	3.72×10^{-06}	0.0	0.84	0.102	0.063	0.102	0.0	-0.100	0.032	-+?	1.78×10^{-03}	14.0

[a] Gene nearest to SNP

[b] A½, effect allele 1 and the other allele 2;

[c] Effect size is relative to the allele 1

[d] Direction of beta of respectively the discovery, ESTHER and Latvia cohorts: positive (+) or negative (-) or not determined (?)

Abbreviations: Chr, chromosome; EAF, effect allele frequency; I^2, measure of heterogeneity; SE, standard error.

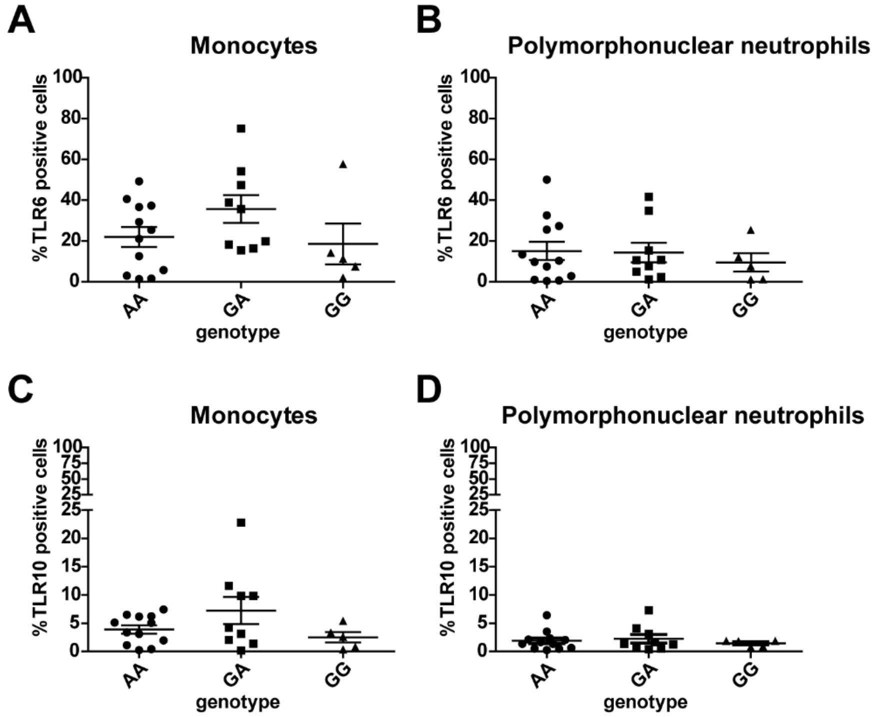

Supplementary Figure S1 Measurement of Toll-like receptor 6 (TLR6) and TLR10-positive cells by flow cytometry. (A-D) Dot plots illustrating the percentage of monocytes and polymorphonuclear neutrophils positive for TLR6 (A-B) or TLR10 (C-D) in healthy subjects (n=26) genotyped for rs28393318: AA (n=12), GA (n=9) and GG (n=5). The mean ±SEM is shown. Dots indicate individual measurements.

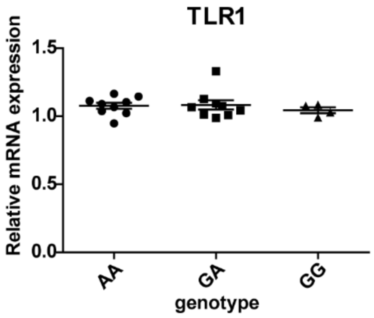

Supplementary Figure S2 Toll-like receptor 1 (TLR1) mRNA expression in blood mononuclear cells (PBMCs). Dot plot representing the relative mRNA expression levels in PBMCs from healthy subjects (n=22) genotyped for rs28393318: AA (n=9), GA (n=9) and GG (n=4). The mean ±SEM is shown, dots indicate individual subjects.

Acknowledgment

The Rotterdam Study (RS) authors are grateful to Prof. dr. Bruno H Stricker and dr. Frank J van Rooij from the department of epidemiology at the Erasmus University Medical Center for providing pharmacological and serological data of RS study participants. The RS authors also acknowledge dr. Wouter J. Den Hollander for his contribution at the initiation of the study.

The MESA authors thank the other investigators, the staff, and the participants of the MESA study for their valuable contributions. A full list of participating MESA investigators and institutions can be found at http://www.mesa-nhlbi.org.

The Latvia cohort authors acknowledge the Genome Database of Latvian Population, the Latvian Biomedical Research and Study Centre for providing data and DNA samples.

References

1. Mayerle J, den Hoed CM, Schurmann C, et al. Identification of genetic loci associated with Helicobacter pylori serologic status. Jama 2013;309:1912-20.
2. Hofman A, Darwish Murad S, van Duijn CM, et al. The Rotterdam Study: 2014 objectives and design update. Eur J Epidemiol 2013;28:889-926.
3. Hofman A, Brusselle GG, Darwish Murad S, et al. The Rotterdam Study: 2016 objectives and design update. Eur J Epidemiol 2015;30:661-708.
4. Ikram MA, Brusselle GGO, Murad SD, et al. The Rotterdam Study: 2018 update on objectives, design and main results. Eur J Epidemiol 2017;32:807-850.
5. John U, Greiner B, Hensel E, et al. Study of Health In Pomerania (SHIP): a health examination survey in an east German region: objectives and design. Soz Praventivmed 2001;46:186-94.
6. Volzke H, Alte D, Schmidt CO, et al. Cohort profile: the study of health in Pomerania. Int J Epidemiol 2011;40:294-307.
7. Dawber TR, Meadors GF, Moore FE, Jr. Epidemiological approaches to heart disease: the Framingham Study. Am J Public Health Nations Health 1951;41:279-81.
8. Feinleib M, Kannel WB, Garrison RJ, et al. The Framingham Offspring Study. Design and preliminary data. Prev Med 1975;4:518-25.
9. Splansky GL, Corey D, Yang Q, et al. The Third Generation Cohort of the National Heart, Lung, and Blood Institute's Framingham Heart Study: design, recruitment, and initial examination. Am J Epidemiol 2007;165:1328-35.
10. Mahmood SS, Levy D, Vasan RS, et al. The Framingham Heart Study and the epidemiology of cardiovascular disease: a historical perspective. Lancet 2014;383:999-1008.
11. Tsao CW, Vasan RS. Cohort Profile: The Framingham Heart Study (FHS): overview of milestones in cardiovascular epidemiology. Int J Epidemiol 2015;44:1800-13.
12. Bild DE, Bluemke DA, Burke GL, et al. Multi-Ethnic Study of Atherosclerosis: objectives and design. Am J Epidemiol 2002;156:871-81.
13. Olson JL, Bild DE, Kronmal RA, et al. Legacy of MESA. Glob Heart 2016;11:269-274.
14. Burke G, Lima J, Wong ND, et al. The Multiethnic Study of Atherosclerosis. Glob Heart 2016;11:267-268.
15. Jaddoe VW, van Duijn CM, van der Heijden AJ, et al. The Generation R Study: design and cohort update 2010. Eur J Epidemiol 2010;25:823-41.
16. Jaddoe VW, van Duijn CM, Franco OH, et al. The Generation R Study: design and cohort update 2012. Eur J Epidemiol 2012;27:739-56.
17. Kruithof CJ, Kooijman MN, van Duijn CM, et al. The Generation R Study: Biobank update 2015. Eur J Epidemiol 2014;29:911-27.
18. Kooijman MN, Kruithof CJ, van Duijn CM, et al. The Generation R Study: design and cohort update 2017. Eur J Epidemiol 2016;31:1243-1264.
19. Low M, Stegmaier C, Ziegler H, et al. [Epidemiological investigations of the chances of preventing, recognizing early and optimally treating chronic diseases in an elderly population (ESTHER study)]Epidemiologische Studie zu Chancen der Verhutung, Fruherkennung und optimierten Therapie chronischer Erkrankungen in der alteren Bevolkerung (ESTHER-Studie). Dtsch Med Wochenschr 2004;129:2643-7.
20. Raum E, Rothenbacher D, Low M, et al. Changes of cardiovascular risk factors and their implications in subsequent birth cohorts of older adults in Germany: a life course approach. Eur J Cardiovasc Prev Rehabil 2007;14:809-14.
21. Schottker B, Saum KU, Perna L, et al. Is vitamin D deficiency a cause of increased morbidity and mortality at older age or simply an indicator of poor health? Eur J Epidemiol 2014;29:199-210.
22. Erglis A, Dzerve V, Pahomova-Strautina J, et al. A population-based cross-sectional study of cardiovascular risk factor in Latvia. Medicina (Kaunas) 2012;48:310-6.
23. Leja M, Shums Z, Nikitina-Zake L, et al. Prevalence estimation of celiac disease in the general adult population of Latvia using serology and HLA genotyping. United European Gastroenterol J 2015;3:190-9.
24. Rovite V, Wolff-Sagi Y, Zaharenko L, et al. Genome Database of the Latvian Population (LGDB): Design, Goals, and Primary Results. J Epidemiol 2018;28:353-360.
